# Hematopoietic stem and progenitors cells gene editing: Beyond blood disorders

**DOI:** 10.3389/fgeed.2022.997142

**Published:** 2023-01-09

**Authors:** Valentina Buffa, José Roberto Alvarez Vargas, Anne Galy, Simone Spinozzi, Céline J. Rocca

**Affiliations:** ^1^ Genethon, Evry, France; ^2^ Integrare Research Unit UMR_S951, Université Paris-Saclay, University Evry, Inserm, Genethon, Evry, France

**Keywords:** gene editing, hematopoietic stem and progenitor cells, inherited disorders, base editing, prime editing, ZFN, TALEN and CRISPR/Cas9

## Abstract

Lessons learned from decades-long practice in the transplantation of hematopoietic stem and progenitor cells (HSPCs) to treat severe inherited disorders or cancer, have set the stage for the current *ex vivo* gene therapies using autologous gene-modified hematopoietic stem and progenitor cells that have treated so far, hundreds of patients with monogenic disorders. With increased knowledge of hematopoietic stem and progenitor cell biology, improved modalities for patient conditioning and with the emergence of new gene editing technologies, a new era of hematopoietic stem and progenitor cell-based gene therapies is poised to emerge. Gene editing has the potential to restore physiological expression of a mutated gene, or to insert a functional gene in a precise locus with reduced off-target activity and toxicity. Advances in patient conditioning has reduced treatment toxicities and may improve the engraftment of gene-modified cells and specific progeny. Thanks to these improvements, new potential treatments of various blood- or immune disorders as well as other inherited diseases will continue to emerge. In the present review, the most recent advances in hematopoietic stem and progenitor cell gene editing will be reported, with a focus on how this approach could be a promising solution to treat non-blood-related inherited disorders and the mechanisms behind the therapeutic actions discussed.

## 1 Introduction

Hematopoietic stem and progenitor cells (HSPCs) are the only cells with the ability to self-renew throughout a person’s lifetime and give rise to all the blood components including leukocytes, platelets and erythrocytes. Rekers and others first explored hematopoietic stem cell transplantation (HSCT) in the fifties, with the demonstration that intravenously injected bone marrow cells prevent the death of irradiated mice by reestablishing blood cell production (Rekers et al., 1950). These studies on animal models were soon translated into clinical applications for patients, especially with the extensive work of the Nobel Prize winner, Dr. E. Donnall Thomas, on allogeneic HSCT (Thomas et al., 1957; Thomas et al., 1977; Thomas et al., 1979), together with the discovery of HLA histocompatibility between donors and recipients (Dausset and Brecy, 1957).

Thus, bone marrow transplantation from healthy HLA-compatible donor has been used to correct genetic disorders such as cytopenias hemoglobinopathies or primary immune deficiencies, by providing HSCs free of pathogenic mutations that can engraft into a myelosupressed host to reconstitute a functional blood and immune system. The procedure of HSC transplantation has also been radically improved by discovering that HSPCs are not only found in high concentrations in the bone marrow, but also in peripheral blood after mobilization (Szade et al., 2018). Although allogeneic HSCT revolutionized medicine even with a HLA match-donor (with the exception of syngeneic twins) and an adapted conditioning protocol, there is always a substantial risk for graft-versus-host-disease (GvHD) in treated patients. GvHD occurs when immune competent T cells in the donated tissue recognize the recipient as non-self. The resulting immune response activates donor T cells to gain cytolytic capacity then attack the recipient to eliminate foreign antigen(s)-bearing cells (Zeiser and Blazar, 2017). . However, as some patients do not have a HLA-compatible donor, the concept of autologous HSCT using *ex-vivo* gene-modified HSPC has been developed by scientists over the years. The first successful attempt of gene therapy using autologous HSCT was performed using gamma retroviral vectors (RVs) in X-linked severe combined immunodeficiency (SCID-X1) and adenosine deaminase deficiency (ADA-SCID) (Bordignon et al., 1995; Cavazzana-Calvo et al., 2000). Unfortunately, the excitement from the promising early results was dampened by the presence of genotoxic events in some of the patients treated with these vectors (Hacein-Bey-Abina et al., 2003; Howe et al., 2008; Cavazzana et al., 2019). Scientists quickly reacted with the development of vectors that allowed robust gene correction in HSPCs, while decreasing insertional mutation events (Galy, 2017). Self-inactivating lentiviral vectors (SIN-LVs) derived from the human immunodeficiency virus (Zufferey et al., 1998) were proved to be more efficient and safer than RVs, and are nowadays the vectors of choice for HSPCs gene therapy, showing long-term benefits in treated patients (Eichler et al., 2017; Kohn et al., 2021; Magnani et al., 2022; Magrin et al., 2022).

More recently, the field of gene and cell therapy has been rattled by the discovery of specific nucleases able to precisely cut genomic DNA. Three main platforms using this strategy exist: Zinc-finger nucleases (ZFNs), Transcription activator-like effector nucleases (TALENs) and Clusters of regularly interspaced short palindromic repeats (CRISPR)/Cas9 nucleases which became the most exploited interface over the past decade. All these nuclease-based approaches rely on the intrinsic ability of the cells to repair double strand breaks (DSBs). . Additionally, the field of gene editing has also seen other tools emerge lately, such as prime editing or base editing (BE), which exploit the specific DNA recognition capacity of CRISPR/Cas9 but use different enzymes to modify the DNA. Most of these tools have already been adopted to gene-modify HSPCs *ex vivo* in an attempt to treat inherited hematopoietic disorders and some studies already led to clinical trials for β-thalassemia and severe sickle cell anemia (Frangoul et al., 2021).

This review will focus on the latest progress for HSPCs gene editing and transplantation, in terms of cells mobilization, selection, culture conditions and the conditioning of patients. We will also give an overview of all the gene-editing tools available and how to efficiently and safely deliver them into HSPCs. And most importantly, we will discuss how gene edited HSPCs could be used not only for blood- or immune-related genetic disorders, but as a vehicle to bring functional proteins to affected tissues in other types of inherited multisystemic disorders.

## 2 Advances in HSPC mobilization, culture and patients conditioning

HSPCs have the ability to reconstitute a new hematopoietic system when transplanted into an immunodepleted patient (Wilkinson et al., 2020). Advances in term of HSPC mobilization and culture together with patient conditioning have been essential to make HSCT safer and more efficient.

### 2.1 Advances in mobilization

HSPCs can be harvested in two ways, by BM aspiration from the pelvic crest or by leukapheresis after induced mobilization into the peripheral circulation. Mobilization is preferred because it is a less stressful procedure and allows for a generally higher stem cell yield, reduced graft failure rates, and faster reconstitution (Gertz, 2010). The recombinant human G-CSF (rh-G-CSF) is the cytokine of choice used for mobilization (Bendall and Bradstock, 2014). This treatment results in a 50–100 fold expansion of the circulating HSPC pool (Holig et al., 2009). In cancer patients, or when higher concentrations of collected HSPCs are necessary, the use of chemotherapy in combination with G-CSF is usually preferred. However, there are major disadvantages linked to the use of chemotherapy, such as toxicity, bone marrow damage and higher costs (Hubel, 2019).

Of note, there are certain conditions where G-CSF cannot be used to mobilize HSPCs, such as sickle cell disease (Fitzhugh et al., 2009). In this particular case, G-CSF can be substituted by the small molecule AMD3100 (Plerixafor) (Esrick et al., 2018). Interestingly, when Plerixafor is combined with G-CSF treatment; it triples the concentration of isolated CD34^+^ cells compared to the administration of G-CSF alone (Liles et al., 2005). Besides the treatments described above, multiple compounds able to increase the number of circulating HSPCs have been evaluated in preclinical models: sphingosine-1-phosphate (Juarez et al., 2012), inducers of MMP-9 (Pelus et al., 2004), or inhibitors of EGFR (Ryan et al., 2010) and Cdc42 (Liu et al., 2019). It has also been reported that treatment with cobalt protoporphyrin (CoPP) induces an endogenous stimulation of cytokines, reaching a better HSPCs mobilization than treatment with G-CSF alone (Szade et al., 2019). In 2018, Hoggatt and others demonstrated that GROβ, a CXCR2 agonist, was able to induce a rapid HSPCs mobilization (within minutes) when used together with the CXCR4 antagonist AMD3100, resulting in a higher engraftment capacity of the isolated cells with respect to those mobilized *via* G-CSF (Hoggatt et al., 2018).

### 2.2 HSPC transplantation without genotoxic conditioning

To ensure the engraftment of transplanted HSPCs, the standard procedure requires a genotoxic conditioning to ablate the endogenous HSPCs and immune system cells, providing the conditions and the “space” that allow for the creation of a donor HSPCs niche and to remove the diseased HSC and progeny. The chemotherapeutic drug commonly used in these settings is busulfan, an alkylating agent with myelosuppressive effects (Bernardo and Aiuti, 2016). However, in spite of low-intensity conditioning regimens which have been developed, there are still negative side effects of busulfan potentially at long term (i.e. organ toxicity, infertility and risk of secondary malignancy), therefore efforts to develop alternative, safer and more specific conditioning regimes have been made (Chanut et al., 2021). The use of specific antibodies against antigens specifically expressed by HSPCs has been the main focus for novel approaches to ablate the resident HSPCs niche. Of particular interest is a study by Czechowicz and others, which showed an almost total depletion of resident HSPCs in immunodeficient mice using ACK2, an antibody that blocks c-kit function (Czechowicz et al., 2007). Similar effects were achieved when using a combination of monoclonal antibodies against c-kit (CD-117) and CD47 (Chhabra et al., 2016), or with antibody–drug conjugates composed of anti-CD117 antibodies and saporin, a ribosome-inactivating protein (Czechowicz et al., 2019). Of note, anti-CD117 conditioning has been already used for allogeneic HSCT in a clinical trial for treating severe combined immunodeficiency (SCID) patients (Agarwal et al., 2020).

Other non-genotoxic conditioning strategies that are of extreme interest have been developed. Pharmacological inhibition of Cdc42 efficiently mobilizes HSPCs from BM in mice, allowing for the engraftment of HSPCs with a better reconstitution potential compared to AMD3100-mediated conditioning (Liu et al., 2019). Interestingly, in a study by Taya and others, removal of dietary valine in mice allowed for host HSPCs depletion supporting efficient engraftment of the transplanted HSPCs (Taya et al., 2016).

### 2.3 *Ex-vivo* expansion of HSPCs

Increasing the number of transplanted corrected HSPCs is determinant for improving the engraftment success rate, especially in settings where there is no selective advantage over the non-corrected HSPCs. A concept confirmed by HSCT clinical trials for Wiskott Aldrich syndrome (Hacein-Bey Abina et al., 2015), or β-thalassemia, where a higher percentage of gene-corrected cells correlated with better outcomes (Thompson et al., 2018). Improving HSPCs viability, function and cell expansion potential, during *ex vivo* manipulation is also a determinant step for increasing engraftment success rates. The Cooke group, discovered a purine derivative named StemRegenin 1 (SR1) able to promote a 50-fold *ex vivo* expansion of CD34^+^ cells, resulting in a 17-fold increase of engrafted cells in immunodeficient mice (Boitano et al., 2010). Another chemical compound of interest is the pyrimidoindole derivative named UM171, which highly promotes human HSC self-renewal and *ex vivo* expansion (Fares et al., 2014; Cohen et al., 2020). HSPCs *ex vivo* expansion *via* three-dimensional culture proved to be also an effective strategy to increase primitive CD34^+^ cells expansion, maintaining a high self-renewal potential and allowing for long-term reconstitution in immunocompromised mice (Bai et al., 2019).

## 3 Gene editing tools

Targetable nucleases can be programmed to induce changes in a specific sequence throughout the genome in a very specific fashion, making them useable gene-editing tools. Examples of these enzymes that have been successfully engineered for use as gene-editing tools in mammalian cells include ZFN, TALENs, and CRISPR/Cas9 system. After the generation of a DSB at a specific locus, the endogenous DSB-repair machinery of the cell can be exploited to insert a new sequence using a donor DNA template. The current genome editing toolbox also includes methods that do not rely on endogenous repair mechanisms, such as prime editors and base editors, which can modify the host’s sequence without induction of DSBs. The intrinsic features of each editing tool may represent strengths or weaknesses depending on the desired application and must be carefully considered when choosing the gene therapy strategy. A description of the currently available gene editing tools and possible advantages and disadvantages in the design of a therapeutic treatment will be briefly discussed in this section.

### 3.1 Zinc-finger nucleases

ZFNs are composed of two zinc-finger domains fused with FokI endonuclease that can be engineered to target a specific genomic region (Kim et al., 1996; Smith et al., 2000). Recognition of the sequence by an individual Zinc finger monomer does not activate the nuclease activity, the DSB is only generated when both zinc-finger domains bind the target sequence and dimerization occurs. Although this feature confers ZFNs a high specificity, the risk for off-target effects is still present (Pattanayak et al., 2011). However, strategies to reduce ZFN off-target effects have been developed, (Liu et al., 2015a; Miller et al., 2019), and despite the design difficulties, ZFNs remain a highly specific tool, depending on the target region (Gabriel et al., 2011; Isalan, 2011).

### 3.2 TALEN

Transcription activator-like proteins (TALEs) are composed by repetitive amino acid (aa) sequences that acts as transcription factors (Bogdanove and Voytas, 2011). TALE nucleases (TALENs) have been engineered as gene editing tools by the fusion of a modular TALE repeats with a FokI domain. Same as ZFNs, dimerization of two TALEN monomers is necessary for FokI activation and DSB generation (Boch et al., 2009; Moscou and Bogdanove, 2009; Cermak et al., 2011; Li et al., 2011; Miller et al., 2011; Gaj et al., 2013). TALENs have also shown a low off-target effects rate in human pluripotent stem cell clones (Veres et al., 2014). However, since TALEs are repetitive sequences that can undergo homologous recombination, their delivery *via* lentiviral vectors is challenging. Additionally, TALENs have affinity to sequences with low G content and the target sequence must begin with a T, substantially restraining the availability of targeting sites (Bogdanove and Voytas, 2011).

### 3.3 CRISPR/Cas

CRISPR/Cas is a naturally occurring genome editing system that bacteria and archaea use as an immune defense against invasive nucleic acids. (Barrangou et al., 2007; Brouns et al., 2008; Sorek et al., 2013). Later, the system was adapted as a gene editing tool by customization of a guide RNA (gRNA) for recognition of the desired genomic sequence and the coupling with high efficient Cas proteins (Jinek et al., 2012). The most commonly used Cas proteins are the SpCas9 (from *Streptococcus pyogenes*) and the Cas12/CpfI (from *Acidaminococcus* and *Lachnospiraceae*) (Cong et al., 2013; Mali et al., 2013; Zetsche et al., 2015). The presence of a protospacer adjacent motif (PAM) sequence immediately downstream of the target site is necessary for effective recognition and cleavage (Hsu et al., 2013; Sternberg et al., 2014). However, CRISPR/Cas9 variants where the PAM sequence presence is dispensable have been developed (Walton et al., 2020; Xie et al., 2022). Addition of a stable hairpin in the gRNA sequence also appears to allow efficient editing regardless of PAM presence in the sequence composition (Riesenberg et al., 2022). For more details about new CRISPR/Cas based tools, please refer to the review by Hu and Li (Hu and Li, 2022).

#### 3.3.1 Base editing

Base editors (BEs) are fusion proteins composed of a catalytically impaired Cas9 (deadCas9 or dCas9) and a single-stranded DNA deaminase, which are able to introduce single-nucleotide variants in the DNA of living cells, without generating DSBs (Komor et al., 2016; Gaudelli et al., 2017; Anzalone et al., 2020). Gene editing strategies that generate DSBs present with a risk for genotoxic events (Kosicki et al., 2018; Cullot et al., 2019; Blattner et al., 2020). Base editing represents a safer option because modification of the DNA sequence occurs without inducing DSBs. Additionally, BE works at any stage of the cell cycle, opposite to gene editing techniques relying on DSBs repair, which are more effective on dividing cells (Zhang et al., 2017). On the other hand, the action of BEs is restricted to the change of a single nucleotide. Although this approach could theoretically treat all diseases characterized by a single point mutation, it is limited by PAM sequence preferences and is restricted by the width of the editing. It is also characterized by high precision, with BEs that can discriminate between the target base and multiple bystander bases within a narrow active window (Jeong et al., 2020). Recent improved versions of classicals BEs are described in the review by Reshetnikov et al. (2022).

#### 3.3.2 Prime editing

Prime editors (PEs) are protein complexes consisting of a PAM-independent Cas9 nickase, a reverse transcriptase and a prime editing gRNA (pegRNA) (Anzalone et al., 2019). PEs present with less off-target activity compared to CRISPR/Cas9, albeit with lower editing efficiency (Liu et al., 2021; Chen et al., 2021; Gao et al., 2022). However, PE-mediated correction of pathologic phenotype has been demonstrated (Zhang H. et al., 2022a; Zhang H. et al., 2022b). Different strategies to improve PEs have been developed, either stabilizing the pegRNA (Liu et al., 2021; Zhang G. et al., 2022), or manipulating repair pathways (Chen et al., 2021; Ferreira da Silva et al., 2022), or a combination of both (Chen et al., 2021; Jiang and Yao, 2022). Furthermore, by inserting specific same-sense mutations in the reverse transcription template of pegRNA or by altering the pegRNA secondary structure, efficiency could be significantly improved (Li et al., 2022).

## 4 Methods of delivery of the gene editing machinery into HSPCs

The main challenge of delivering of gene editing tools together with the corrective DNA template into HSPCs *ex vivo* or *in vivo* is obtaining HSPCs editing in the most efficient and safe manner to procure HSPC’s progeny with the corrected DNA information. To avoid toxicity and off-target activity, the enzyme needs to be optimized to create a specific DSB in the shortest possible period. The choice of carrier depends on the repair pathways the cells will be using during the editing process, the level of editing that needs to be achieved, the toxicity tolerance of the treated cells and, in the case of homology-directed based approaches, the size of donor template (Table 1).

**TABLE 1:** Delivery method comparison for gene editing.

Carrier/delivery method	Delivered material	Advantages	Disadvantages	References
Electroporation	Plasmid DNA/mRNA/RNP/ssODNs	- high transfection efficiency - enable transfection of difficult cells - reduction of off-target events - reduction insertional mutagenesis - low immunogenicity (RNP) - viral free	- restricted to *ex vivo* application - cell toxicity	(Urnov et al., 2005; Hendel et al., 2015; Liu et al., 2015; DiGiusto et al., 2016; Gundry et al., 2016; Chandrasekaran et al., 2018; Roth et al., 2018; Vakulskas et al., 2018; Conway et al., 2019; Lattanzi et al., 2019; Lux et al., 2019; Rocca et al., 2020; Bloomer et al., 2021)
LNPs	mRNA	- *in vivo* application - viral free - low cost	- difficulty to target HSPCs - toxicity concerns	(Sago et al., 2018a; Sago et al., 2018b; Cannon et al., 2021)
Adenovirus vectors (AdVs)	dsDNA	- *in vivo* application - enables simultaneous packaging of CRISPR components - high packaging capacity (37 kb) - low cost for vector manufacturing - episomal expression - absence of viral gene expression - transduce non-dividing cells (HDAd5/35++)	- immunogenicity - only the serotype HDAd5/35++ shows affinity to HSPCs	(Li et al., 2013; Xu et al., 2017; Li et al., 2021)
adeno-associated virus vectors (AAVs)	ssDNA	- *in vivo* application - high transfection efficiency - non-pathogenic - infection of non-dividing cells - do not integrate in the host genome - serotype 6 has high affinity for HSPCs - low immunogenicity - mainly used to bring DNA donor Template	- low packaging capacity (4.8 kb) - persistence in targeted tissue - can trigger P53 Response	(Rai et al., 2020; Yang et al., 2020; Brault et al., 2021; Cromer et al., 2021; Wilkinson et al., 2021)
Self-inactivated lentiviral vectors (SIN-LVs)	ssRNA	- large cargo capacity (10 kb) - high transfection efficiency - enables simultaneous packaging of CRISPR components - infect both dividing and non-dividing cells - broad tissue tropism - no expression of viral proteins after vector transduction - safer integration site profile	- potential insertional mutagenesis - off-target effects due to persistent expression	(Miyoshi et al., 1998; Zufferey et al., 1998; Sakuma et al., 2012)
Integrative-deficient LVs (IDLVs)	ssRNA or dsDNA (donor template)	- large cargo capacity infect both dividing and non-dividing cells - do not integrate into host genome - vehicle to bring the DNA donor template	- persistence in targeted tissue	(Philippe et al., 2006; Yanez-Munoz et al., 2006; Lombardo et al., 2007; Abdul-Razak et al., 2018)
Vector Like Particles (VLPs)	- RNP (Nanoblades, eVLPs) -Cas9 mRNA (LVLPs)	- *in vivo* application - DNA free (eVLPs) - reduction of off target risk	- low efficiency	(Lu et al., 2019; Mangeot et al., 2019; Banskota et al., 2022)

### 4.1 Physical delivery of gene editing tools into HSPCs

Mobilization and isolation of HSPCs allowing their *ex vivo* manipulation offers a clear advantage for genetic manipulations. Compared to DNA vectors, mRNA increases the transfection efficiency of inactive cells, does not present the risk of insertional mutagenesis in the host’s genome, allows for a faster protein production with higher expression levels control, and does not contain additional exogenous sequences, such as antibiotic resistances or viruses-derived promoters (Guan and Rosenecker, 2017; Baptista et al., 2021). A lot of efforts have also been deployed to improve stability and translational efficiency of the mRNA filament (Korner and Wahle, 1997; Jemielity et al., 2003; Gustafsson et al., 2004; Kwon et al., 2018). as well as reducing the potential immunogenicity of transcribed mRNA*in vitro *(Triana-Alonso et al., 1995; Wu et al., 2020) (Kariko et al., 2008; Andries et al., 2015) (Mu et al., 2018) (Vaidyanathan et al., 2018) *in vitro *(Nelson et al., 2020). Delivery of transcribed mRNA coding for ZFN (DiGiusto et al., 2016; Conway et al., 2019), TALEN (Lux et al., 2019) and CRISPR-Cas9 (Hendel et al., 2015) into HSPCs showed great promises in terms of efficacy and safety. Finally, nucleases can also be delivered directly as proteins (Liu et al., 2015b), providing rapid action and fast turnover, hence reducing off-target effects. Nowadays, the gold standard to efficiently and safely perform CRISPR-Cas9 gene editing in HSPCs is nucleofecting both, the mRNA (guide-RNA) and the nuclease (Cas9) as pre-assembled ribonucleotide-protein complexes (RNP) (Gundry et al., 2016; Vakulskas et al., 2018; Lattanzi et al., 2019; Rocca et al., 2020; Bloomer et al., 2021). In addition, transient genome editing, reduces off-target effects, insertional mutagenesis, and immune responses, besides allowing for high editing efficiencies (Chandrasekaran et al., 2018).

### 4.2 Chemical delivery into HSPCs

Chemical delivery of gene editing tools is only of interest for *in vivo* delivery into HSPCs (not achievable by electroporation). In some cases, the patient’s condition might be too severe to consider a myeloablative treatment followed by HSCT, or the opposite, a condition too mild to consider such an invasive procedure. A chemical approach could also significantly reduce costs of HSPC-based therapies, making it more accessible to less privileged populations.

Recently, a c-kit nanoparticle that enables a partial *in vivo* targeting of HSPCs has been developed (Cannon et al., 2021). Another promising approach is the use of lipid nanoparticles (LNPs) for mRNA delivery to target cells *in vivo* (Mukai et al., 2022). LNPs are known to have high affinity for the hepatic tissue, but their surface can easily be crosslinked to antibodies to redirect their cellular specificity for the desired *in vivo* targeting (Rosenblum et al., 2020; Tombacz et al., 2021). The Dahlman laboratory developed a method using barcoding and bioinformatics to study biodistribution of LNPs *in vivo* and selected a LNP named BM1 able to target bone marrow endothelial cells (Sago et al., 2018a; Sago et al., 2018b). Finally, it has been reported by Weissman and collaborators that a CD90-targeting LNPs can transfect about 4% of CD34^+^ cells from bone marrow stem cell preparations (Cannon et al., 2021).

### 4.3 Viral and “viral-like” delivery into HSPCs

Different viral platforms that can be used to bring nucleases and donor template into HSPCs: Adenoviral vectors (AdVs), adeno-associated viral vectors (AAVs) and lentiviral vectors (LVs). The choice of the viral vector type depends on the size of the genetic material to carry and on the virus ability to transduce either quiescent or dividing cells.

AdVs are double-stranded DNA viruses capable of packaging up to 37 kb of DNAExtensive studies trying to optimize helper-dependent AdVs for transducing CD34^+^ have been performed not only because of their large packaging capacity, but also for the low cost for vector manufacturing, the episomal expression and the absence of viral gene expression (Li and Lieber, 2019). Based on the observation that HIV patients had non-detectable viral levels after HSCT with donor cells homozygous for CCR5delta32 (Hutter et al., 2009; Gupta et al., 2019), the targeting of HSPCs using AdVs able to inactivate CCR5 using ZNF or CRISPR/Cas9 have been the focus of several studies in the context of HIV-1/AIDS therapies (Li et al., 2013; Xu et al., 2017). In a recent study, the Lieber laboratory reported a novel approach to treat β-Hemoglobinopathies in CD46/β-YAC transgenic mice using AdVs and a base editing approach (Li et al., 2021).

AAVs have the advantages of being non-pathogenic, to efficiently infect non-dividing cells and, they do not integrate in the host genome. The main limitation of AAVs is their low packaging capacity (4.8 kb). However, this can be improved with the development of dual-AAV donor vector systems (Bak and Porteus, 2017). Serotype 6 is the AAV with the higher tropism for HSPCs (Yang et al., 2020). Currently, most of gene editing approaches for therapeutic protein expression need the usage of both gene editing tools and AAV6 (Rai et al., 2020; Brault et al., 2021; Cromer et al., 2021; Wilkinson et al., 2021).

LVs possess several advantages for HSPC manipulation: a large cargo capacity (∼10 kb), a stable integration into host genome, they can infect both dividing and non-dividing cells, no expression of viral proteins after transduction, the ability to deliver complex genetic elements, and a safer integration site profile (Sakuma et al., 2012). Researchers applied several genetic modifications over the years to make LVs safer for gene therapy, leading to the development of self-inactivating LVs (SIN-LVs) (Miyoshi et al., 1998; Zufferey et al., 1998) and integrative-deficient LVs (IDLVs) (Philippe et al., 2006; Yanez-Munoz et al., 2006). IDLVs have been successfully used for ZFNs delivery to the target cells and the corrective DNA template to HSPCs in the context of PIDs (Lombardo et al., 2007; Abdul-Razak et al., 2018).

Although AAV6 and IDLVs are powerful platforms to deliver DNA donor templates, both can persist in targeted tissue for long periods and may lead to unwanted effects (Cousin et al., 2019; Bolt et al., 2021). As an alternative, single-stranded oligodeoxynucleotides (ssODNs) can be used (short oligos <200bp with 30-60bp homology harms). They showed great potential for CRISPR/Cas9/HDR in HPSCs (Antony et al., 2018; Romero et al., 2019), having though the disadvantage of only permitting small insertion changes (point mutations or short fragments), however the technology is improving extremely fast (Roth et al., 2018).

To deliver gene-editing tools *in vivo*, a research group developed a viral-like approach named nanoblades, based on HIV or murine leukemia virus. The strategy consists in generating virus-like particles with the Gag protein fused to Cas9 and gRNA expression cassettes (Mangeot et al., 2019). After, these particles are pseudotyped with glycoproteins to specifically target HSPCs (Mangeot et al., 2019; Gutierrez-Guerrero et al., 2021). In 2019, the Atala laboratory developed a system able to package up to 100 copies of *Staphylococcus aureus Cas9* (*SaCas9*) mRNA in each LV-like bionanoparticle (LVLP) (Lu et al., 2019). Finally, the Liu laboratory reported the development of DNA-free virus-like particles (eVLPs) able to efficiently package and deliver base editor or Cas9 ribonucleoproteins to different cell types using different glycoproteins (Banskota et al., 2022).

## 5 Methods to improve HDR-mediated gene editing in HSPCs

DNA DSBs generated by editing nucleases can be repaired *via* different pathways in mammalian cells depending on the cell cycle phase but also on the DNA end structures induced by specific programmable nucleases (*i.e.,* blunt or cohesive ends). Quiescent HSPCs rely mainly on the NHEJ pathway to repair DSBs, because it is the only one operating during the G0/G1 phases of the cell cycle. However, this pathway is not homology dependent and can lead to sequence modifications at the repair junctions. On the other hand, MMEJ is active during the S/G2 phases of the cell cycle but can generate even larger indels with respect to NHEJ. In presence of a DNA template, targeted integration is possible with both NHEJ and MMEJ, but these are still not error-free alternative pathways. Single-strand annealing (SSA) and homologous recombination (HR) also occur during S/G2. . HR is considered error-free and consequently it is the DBS repair pathway of choice for gene editing. However, as mentioned earlier, HSPCs are quiescent cells and HR frequency is very low in these cells. In this chapter, we are going to review the most recent strategies developed to trick this biological system and increase homologous directed repair (HDR) frequency in HSPCs.

### 5.1 Choice of DNA donor template

The choice of delivery method of the donor DNA template alone (i.e. IDLVs, AAV6 or ssODNs) has been shown to influence HDR frequency in HSPCs. Several studies showed that AAV6 allows for higher HDR rates in HSPCs, independently of the nuclease used (Wang et al., 2015; Dever et al., 2016; Schiroli et al., 2017; Kuo et al., 2018; Pavel-Dinu et al., 2019; Rai et al., 2020; Bijlani et al., 2021). It is known that ssODNs are the preferred substrates for the single-stranded template repair (SSTR) pathway, rather than the conventional HDR and preferably used to correct few nucleotides (Richardson et al., 2018). Pattabhi and others showed that even though AAV6 were more efficient *in vitro* with respect to ssODN, a higher proportion of ssODN-modified cells persisted *in vivo* (Pattabhi et al., 2019).

### 5.2 Addition of molecules

Salisbury-Ruf and others have recently reviewed an extensive list of molecules used to modulate HDR in mammalian cells (Salisbury-Ruf and Larochelle, 2021). Briefly, they classified these modulators in five categories: inhibitors of NHEJ proteins, promoters of HDR, modulators of the cell cycle, molecules targeting chromatin structure, and molecules with undefined mechanisms (Salisbury-Ruf and Larochelle, 2021). *Ex vivoex vivoin vivo*. Interestingly, when using IDLVs to bring the donor DNA template, transduction in presence of the immunosuppressive molecule cyclosporine H considerably enhances HDR efficiency. Cyclosporine H inhibits the interferon-induced transmembrane protein 3 (IFITM3), responsible for hampering VSV glycoprotein-mediated vector entry (Petrillo et al., 2018).

### 5.3 Expression of proteins

Addition of proteins that influences gene integration at the DSB locus towards HDR in HSPCs have also been investigated. In 2016, Gutschner and others developed a strategy to increase HDR by directly synchronizing the expression of Cas9 during the S/G2 phase fusing Cas9 to the N-terminal region of human Geminin (Gutschner et al., 2016). This hGemCas9 construct has also been evaluated in HSPCs by D. Khon and others for targeting of the *HBB* gene. In this study a 4-fold increase in HDR/NHEJ ratio in primary human HSPCs *in vitro* was obtained and a significant improvement in HDR/NHEJ ratio *in vivo* (Lomova et al., 2019). In 2019, Schiroli and others showed that transient expression of the dominant-negative P53 mutant protein GSE56 during editing increased hematopoietic repopulation (Schiroli et al., 2019). In 2020, the same group combined GSE56 with expression of the protein E4orf6/7, which recruits the cell cycle controller E2F on its target genes. They showed that with this combined strategy, HDR editing efficiencies of up to 50% in the long-term graft were obtained, without perturbing the function of edited HSPCs (Ferrari et al., 2020). In 2021, De Ravin and others showed that i53, an engineered ubiquitin variant with a high affinity to the tandem Tudor domain of 52BP1, effectively reduces NHEJ repair, and promotes HDR in CD34^+^ HSPCs (De Ravin et al., 2021). Finally, other proteins tested in non-HSPCs cellular models showed great potential in increasing HDR efficiency, such as CtIP (Charpentier et al., 2018), AUNIP (Lou et al., 2017) or Pold-3 (Reint et al., 2021).

### 5.4 Additional strategies: Selection of corrected cells and bypassing HDR

Increasing HDR is not the only way to obtain an enriched population of edited cells. Dever and others were able to achieve 90% HBB-edited HSPCs in long-term graft by embedding a reporter cassette in the HDR template (Dever et al., 2016). Another research group designed a robust co-selection strategy using CRISPR–Cas9 and CRISPR-Cpf1 to generate dominant cellular resistance to ouabain and simultaneously edit a second locus of interest (Agudelo et al., 2017). Another possibility to improve editing frequency in HSPCs is the exploitation of the NHEJ pathway to bypass the inefficiencies of HDR-mediated approaches (Bloomer et al., 2021) (Roman-Rodriguez et al., 2019).

## 6 HSPC gene editing in multisystemic disorders

**FIGURE 1:**
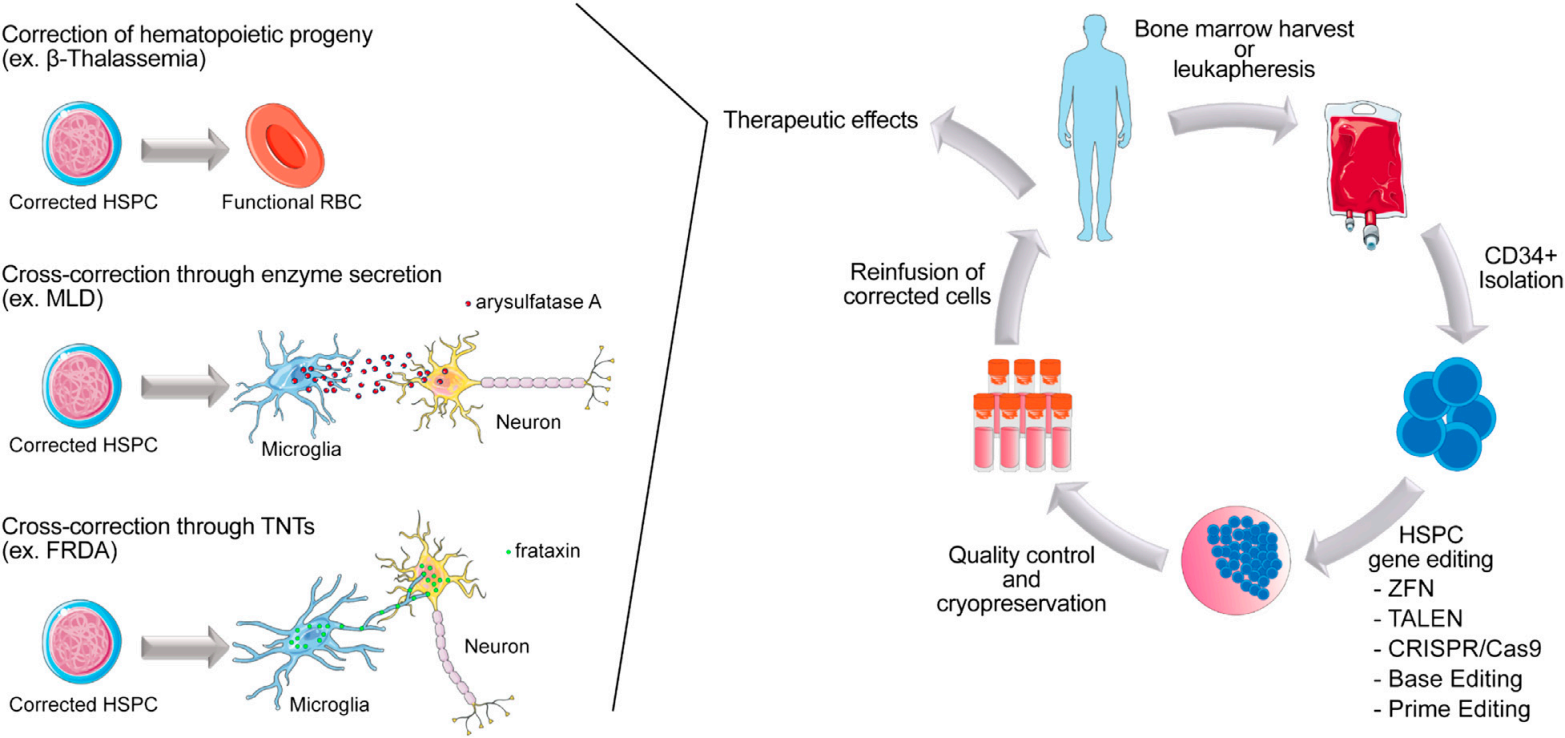
Scheme of autologous HSPC gene editing in patients affected by inherited genetic disorders. HSPCs are collected from the patient from the bone marrow or peripheral blood. CD34^+^ are isolated and gene modified *ex vivo*, using gene editing tools. After a short period of culture, cells are cryopreserved before being reinfused in patients. Once engrafted, corrected HSPCs can give rise to functional blood and immune cells in the context of blood related disorders such as Beta-thalassemia. Corrected HSPCs can also cross the blood brain barrier and differentiate into microglia to perform tissue cross correction by either enzyme secretion (ex. MLD) or protein transfer *via* TNTs (ex. FRDA).

Nowadays, the portfolio of gene editing tools available and all the progress made in HSPCs processing methods provided great insights for the development of less toxic and more precise novel applications. As a result, during the last decade, multitudes of preclinical studies combining autologous HSCT and gene editing tools for treatment of various disorders were conducted. Diseases due to hematopoietic-derived cells/tissue alterations are the main target for this kind of therapy, and studies targeting blood and immune disorders have been extensively reviewed elsewhere (Jensen et al., 2019; Antoniou et al., 2021; Ferrari et al., 2021; Rai et al., 2021). In this part of the present manuscript, the focus will be on the ability of HSPC-derived cells in communicating with their surrounding environment to receive and pass on information through different mechanisms, such as direct cellular contact and/or secretion of proteins. We will review these different ways of communication and how researchers take advantage of them to develop therapeutic approaches for multisystemic genetic disorders using gene-editing HSPC (Figure 1).

### 6.1 Tissue repair mediated by secretion of therapeutic proteins from gene corrected HSPC-derived cells to diseased tissues

From different successful attempts to cure non-hematologic genetic disorders by allogeneic HSCT, it has been demonstrated that HSPC-derived cells are able to secrete curative proteins. This is particularly true in the context of congenital metabolic diseases (Steward and Jarisch, 2005). In 2004, Biffi and others. demonstrated that the progeny of *ex vivo* LV-transduced HSPCs transplanted in a mouse model for the neurodegenerative disorder metachromatic leukodystrophy (MLD) were present in the central and peripheral nervous system, where these cells expressed and secreted arylsulfatase A, rescuing the neurodegenerative phenotype. They showed that *ex vivo* gene therapy had a significantly higher therapeutic impact compared to allogeneic transplantation of WT HSPCs, indicating a critical role for enzyme overexpression in the HSPCs progeny (Biffi et al., 2004). In 2006, the same group showed that efficacy of this approach correlated with the overexpression of arylsulfatase A in the microglia progeny (Biffi et al., 2006). Similarly, in 2010, Visigalli and others showed that autologous HSCT could rescue the mucopolysaccharidosis type I (MPS-I) phenotype in a mouse model for the disease. In this study, lentiviral-based HSCT resulted more effective with respect to allogeneic HSCT, thanks to supranormal enzyme activity (α-l-iduronidase) in the lentiviral transduced hematopoietic system of the transplanted mice, allowing for enzyme delivery to the brain and skeleton and disease correction (Visigalli et al., 2010a). In 2020, Piras and others obtained similar results in a murine model for Pompe Disease. They observed phenotypic correction of heart and muscle function in mice transplanted with lentiviral-based gene modified HSPCs overexpressing the acid alpha-glucosidase enzyme (GAA) (Piras et al., 2020). Numerous studies have demonstrated similar results when autologous HSPCs were transduced with lentivirus to obtain an elevated production of the enzyme of interest in the progeny. In these settings, HSPC-derived cells become a “vehicle” that secretes the functional enzyme in concentrations high enough to compensate the complete absence of enzymes that is normally produced by other cell types (Yoshimitsu et al., 2007; Sergijenko et al., 2013; Adhikari et al., 2021; Dahl et al., 2021).

Although autologous HSCT became one of the most valuable options for patients with particular congenital metabolic diseases (Tucci et al., 2021), risks associated with the use of lentiviral vectors still remain, as previously discussed. Similar to other inherited disorders, also in congenital metabolic diseases gene editing at the specific genomic location has been investigated as an option to overcome these potential risks. In 2019, the Porteus laboratory presented an efficient *ex vivo* genome editing approach using CRISPR-Cas9, targeting α-l-iduronidase to the CCR5 safe harbor locus in human HSPCs. The modified cells secrete supra-endogenous enzyme levels, maintain long-term repopulation and multi-lineage differentiation potential and can improve biochemical and phenotypic abnormalities in an immunocompromised mouse model of MPS-I (Gomez-Ospina et al., 2019). The exact same approach, but overexpressing the glucocerebrosidase, was used in 2020 by Scharenberg and others as a potential treatment for Gaucher disease. In this study the authors also used a monocyte/macrophage promoter to restrict expression to this carrier cell type (Scharenberg et al., 2020). In a very interesting study, Pavani and others developed a system to highly express therapeutic enzymes in HSPC-derived erythroblasts. The genes for α-globin expression are present in four copies per cell and loss of up to three α-globin alleles is mostly asymptomatic, making this locus a promising candidate for knock-in (KI) in HSPCs using AAV6 and CRISPR/Cas9. Exploiting this notion, they demonstrated that KI of three different human transgenes encoding for α-l-iduronidase (Hurler syndrome), or α-galactosidase (Fabry disease), or lysosomal acid lipase (Wolman disease) at the α-globin locus was possible and resulted in a substantial increase of enzyme expression upon erythroid differentiation (Pavani et al., 2020). Finally, Anthony and others proposed a mutation-agnostic HSPC gene therapy using CRISPR-Cas9 and AAV6 repair template as a prospective treatment option for MLD. In this fascinating study, enzyme activity in HSPC’ patients was restored to levels similar to healthy adults, after gene editing (Antony et al., 2022).

### 6.2 Tissue repair mediated by protein transfer from gene corrected HSPC-derived cells to diseased tissues *via* tunneling nanotubes

Tunneling nanotubes (TNTs) have first been described *in vitro* as highly sensitive nanotubular *de novo* formed structures that create complex networks and facilitate the selective transfer of membrane vesicles and organelles between cells (Rustom et al., 2004). Later, the presence of TNTs have also been reported *in vivo* in bone marrow-derived MHC class II(+) cells of the corneal stroma (Chinnery et al., 2008), between human monocyte-derived macrophages (Onfelt et al., 2006) and between T cells as a novel route for HIV-1 transmission (Sowinski et al., 2011). After extensive studies, TNTs are currently defined as cytoplasmic bridges between two close or distant cells. They form gap-like junctions between connected cells and mediate the exchange of cytoplasmic proteins, cellular organelles, lipids, nucleic acids, microRNA, ions and several other components (Lou et al., 2012; Jackson et al., 2016; Drab et al., 2019). Only thicker TNTs containing microtubules (>7-µm diameter) allow for transfer of organelles like mitochondria (Eugenin et al., 2009; Jackson et al., 2016). They facilitate short and long-distance cell-to-cell direct communication (up to 300 µm) (Dubois et al., 2020). TNTs are able to polymerize and depolymerize rapidly (30–60 s), property that makes them fluid and transient structures (Gerdes et al., 2013). TNTs are involved in several pathologies (Tiwari et al., 2021) and generate from different cell types, including multiple HSPCs-derived immune cells (Onfelt et al., 2004).

One of the most powerful features of HSPC-derived cells is the ability to cross the blood brain barrier, hence facilitating the success of the treatment in neuromuscular diseases (Lampron et al., 2012). In 2017, we demonstrated that allogeneic healthy HSCT could rescue the phenotype in a mouse model for Friedreich’s ataxia (FRDA), a neurodegenerative disorder caused by GAA triplets expansion in the *FXN* gene (Rocca et al., 2017). Our data showed that engrafted HSPC-derived microglia/macrophages were able to transfer the functional mitochondrial frataxin protein to neurons, muscle fibers and cardiomyocytes of the transplanted FRDA mice, preventing the development of motor defects (Rocca et al., 2017). In the same study, we also evidenced the mechanism by which HSPC-derived microglia/macrophages cross-correct affected tissues. These cells transfer mitochondria containing the functional frataxin to neighboring diseased cells by direct contact *via* TNTs (Rocca et al., 2017). In a more recent manuscript, we provided the foundation for clinical translation of autologous transplantation of gene-corrected HSPCs as a treatment for FRDA (Rocca et al., 2020). Using RNP composed of two gRNAs surrounding the pathologic GAA(n) region and the Cas9 protein, we efficiently removed the pathologic intronic GAA expansion in CD34^+^ cells isolated from FRDA patients’ peripheral blood, and demonstrated that gene correction restored frataxin expression, allowed for normal hematopoietic differentiation and mitochondrial function in treated cells, *in vitro* and *in vivo* (Rocca et al., 2020).

The ability of HSPCs to engraft tissues and differentiate into macrophages/microglia to transfer a functional protein *via* TNTs have been used as a therapeutic approach also in other genetic disorders. The Cherqui laboratory has pioneered the use of HSPCs to transfer lysosomal proteins *via* TNTs in the lysosomal storage disorder Cystinosis (Harrison et al., 2013; Naphade et al., 2015; Rocca et al., 2015; Gaide Chevronnay et al., 2016; Rocca and Cherqui, 2019). The Devuyst laboratory successfully applied this approach in a mouse model of Dent disease (Gabriel et al., 2017). Overall, these results open new perspectives for the development of novel gene-modified HSPCs-based therapies for genetic disorders in which the dysfunctional protein is expressed in cellular organelles like lysosomes or mitochondria.

### 6.3 The strange case of X-linked adrenoleukodystrophy

X-linked adrenoleukodystrophy or X-ALD is a severe genetic demyelinating disease caused by a deficiency of ALD protein, an adenosine triphosphate-binding cassette transporter encoded by the ABCD1 gene. Although the mechanism of rescue is unclear, allogeneic and autologous lentiviral-based HSCT have shown to be effective in the treatment of X-ALD when performed at an early stage of disease (Cartier et al., 2009; Cartier and Aubourg, 2010; Cartier et al., 2012; Eichler et al., 2017). In X-ALD, the mutated gene ABCD1 encodes a peroxisomal membrane protein that cannot be secreted. The invasion of macrophages and/or cross-trafficking of monocytes have been suggested as a possible mechanism. Theory based also on the fact that inflammatory demyelination and microglial cell death “create space” for long-term repopulation of the brain parenchyma with residential macrophages/microglia derived from HSPCs (Cartier and Aubourg, 2010; Varvel et al., 2012; Hohsfield et al., 2020). On the other hand, Yamada and others demonstrated that cell-to-cell contact between healthy microglial cells and ALD fibroblasts was necessary for a phenotypic rescue *in vitro*, suggesting that cell replacement might not be the only mechanism (Yamada et al., 2004).

Although, gene editing in X-ALD HSPCs has not been investigated yet, the correction of an ALD patient-derived iPSC model using ssODN and the CRISPR/Cas9 system has been reported in a recent study, where the cell line exhibited normal iPSC pluripotency marker expression following genome editing (Sik Jung et al., 2022).

### 6.4 Challenges of autologous HSCT in treating non-blood disorders

Treating non-blood genetic diseases with genetically modified autologous HSPCs could present with additional challenges. For inherited blood disorders, gene therapy correct the expression of a protein that is naturally expressed in the hematopoietic line. On the contrary, in non-blood genetic disorders HSPC derived cells are used as vehicles to bring a functional protein that in physiological settings is produced by other cell types. With this approach, the integration of the transgene and the expression of a “non-physiological” protein in the HSPCs could lead to several issues: difficulties to control the expression of the protein, impact on the transplanted HSPCs niche itself and/or on the HSPC differentiation potential into blood cells. This is particularly true for metabolic disorders where high levels of enzymes are needed to achieve efficacy. A good example has been provided by Visigalli and others, which demonstrated that a supraphysiologic galactocerebrosidase activity, an enzyme that is dysfunctional in the lysosomal storage disorder globoid cell leukodystrophy, is associated with functional abnormalities affecting HSPCs and their niche (Visigalli et al., 2010b). However, most of these issues are theoretically avoided when using gene editing to restore expression of a dysfunctional protein. In this case, either the gene is corrected but still under the control of its own promoter, or a transgene is integrated under the control of an existing promoter, hence limiting the impact on the HSPCs physiology.

## 7 Final note

A lot of progress in the field of HSPC-based gene editing has been made in the last decade. Thanks to the significant amount of data obtained from studies and clinical trials involving HSPC in gene therapy, scientists have strong basis to improve existing methods and develop novel approaches more rapidly and safely. In this manuscript, we reviewed some advancements made in HSPC mobilization, culture and patient conditioning. We discussed how gene editing tools have become more and more specific and the various methods to safely deliver them. Most importantly, we also described the range of applications for this approach. HSPCs can be edited *ex-vivo* not only to replace a specific hematopoietic derived cell type or to re-create a diseased hematopoietic system, but also to use them as a vehicle to deliver a missing or dysfunctional protein in an inherited pathological setting. The various limitations associated to editing-based HSCT has already been extensively reviewed elsewhere (Ferrari et al., 2021; Koniali et al., 2021). However, it is important to mention that the main obstacles associated to gene editing of HSPCs for clinical applications are: a) The risks associated to off-target activity generated by the enzymes and consequently the need to develop more sensitivity and complementary techniques to detect them *ex-vivo* but also in the long-last graft in patients. b) The problems associated to the DSB toxicity. c) The issues associated with immunogenicity, although this is more a concern for *in vivo* applications. Finally, another main concern for clinical applications is the cost and access to this type of therapy.
